# Chronic traumatic encephalopathy in a former Australian rules football player diagnosed with Alzheimer’s disease

**DOI:** 10.1186/s40478-020-0895-z

**Published:** 2020-02-26

**Authors:** Alan J. Pearce, Joanne Sy, Maggie Lee, Antony Harding, Rowena Mobbs, Jennifer Batchelor, Catherine M. Suter, Michael E. Buckland

**Affiliations:** 1 College of Science, Health and Engineering, La Trobe University, Bundoora, VIC 3086 Australia; 2 Department of Neuropathology, Royal Prince Alfred Hospital, Camperdown, NSW 2050 Australia; 3 Brain & Mind Centre, University of Sydney, Camperdown, NSW 2006 Australia; 4 Macquarie University, Macquarie Park, NSW 2109 Australia

**Keywords:** Chronic traumatic encephalopathy, Traumatic brain injury, Australian football league, Concussion, Repetitive head injury, Dementia, Neurodegeneration, Tau, Public health, Occupational health

**To the Editor:**

The first case report of chronic traumatic encephalopathy (CTE) in a National Football League player in 2005 [9] opened the floodgates for the identification of CTE in American football. CTE is now reported in ex-players of other contact sports, including ice hockey, soccer, rugby union, and most recently in Australian rugby league [2]. To date, repetitive head injury remains the only known risk factor for the development of CTE [3]. Here we describe the first case of CTE in Australian rules football (ARF), the most popular contact sport in Australia.

The decedent was a male in his 9th decade who had played more than 350 first-grade matches of ARF over 19 years. At age 64 he was diagnosed with Alzheimer’s disease (AD), with accompanying personality change, depression and anger/aggression issues around this time. He had been diagnosed with REM sleep behaviour disorder several years prior to his presumptive AD diagnosis. His cognitive issues were dominated by memory loss, which was slowly progressive until a distinct acceleration in the last ~ 5 years of life. Mild Parkinsonian features of uncertain aetiology were identified several years after his AD diagnosis, possibly related to low-dose antipsychotic medication. He also had intercurrent ischaemic heart disease, hypercholesterolaemia, and hypertension, all of which were well managed. He did not use alcohol, tobacco, or illicit drugs.

Table 1 summarises the relevant neuropathology. There was mild-moderate frontal and temporal lobe atrophy with *ex-vacuo* ventriculomegaly (lateral and third ventricles), mild uncomplicated atheroma in the basal vasculature, and pallor of the substantia nigra. Phosphorylated Tau immunoreactivity (pTau) was present in many grey matter regions. Neocortical pTau was markedly concentrated in an irregular perivascular distribution at sulcal depths in the soma and processes of both neurons and astrocytes: this is the defining lesion of CTE [8] (Fig. 1a, b). Twelve CTE foci were present within nine frontal lobe blocks, and four foci in four temporal lobe blocks. In the temporal and insular cortices there was also dense involvement of superficial layers (layers II-III) (Fig. 1c), consisting of pretangle and tangle pTau, and some ghost tangles. This pattern of pTau deposition, commonly seen in severe CTE, is distinct from the typical pTau deposition in AD (Fig. 1d). Neuronal pTau was composed of both 3R and 4R isoforms, while astrocytic pTau was predominantly 4R.

**Table 1:** Summary of neuropathology findings

Tau pathology	
Depths of cortical sulci, perivascular *neuronal, astrocytic, neuritic*	frontal +++ temporal ++ parietal, occipital -
Prominent superficial neocortical layers *neuronal, astrocytic, neuritic*	temporal +++ insular +++ frontal + parietal, occipital -
Hippocampus *neuronal*	CA2, CA4 +++ CA3 ++ DG + CA1 sclerosis (*astrocytic*) +
Amygdala *neuronal, neuritic*	+++
Striatum, Lentiform nuclei *neuronal, neuritic*	+
Thalamus *neuronal, neuritic*	+
Hypothalamus incl. Mammillary body *neuronal, neuritic*	+++
Midbrain *neuronal, neuritic*	substantia nigra +++ median raphe +++ tectum ++
Pons	locus coeruleus + abducens nucleus +
Medulla	–
Cerebellum	–
Subpial & periventricular ARTAG	present
Ghost tangles	CA1, entorhinal, superficial temporal, amygdala
pTDP-43 pathology *NCI, neuritic*	amygdala ++ hippocampus + superficial temporal ++ depths of frontal sulci +
Other pathology	
Vascular disease, arteriolosclerosis Vascular disease, atherosclerosis	basal ganglia +++ subcortical white matter +++ basal vessels +
Beta-A4 (amyloid)	Thaal 4 (A3)
CERAD score	C2
Alpha-synuclein	absent
Diagnosis	**CTE Stage III** **AD-NC (A3, B2, C2)**

*CA* cornu ammonis, *DG* dentate gyrus, *NCI* neuronal cytoplasmic inclusions, *AD-NC* Alzheimer’s Disease neuropathologic change, *ARTAG* aging-related tau astrogliopathy

**Fig. 1:**
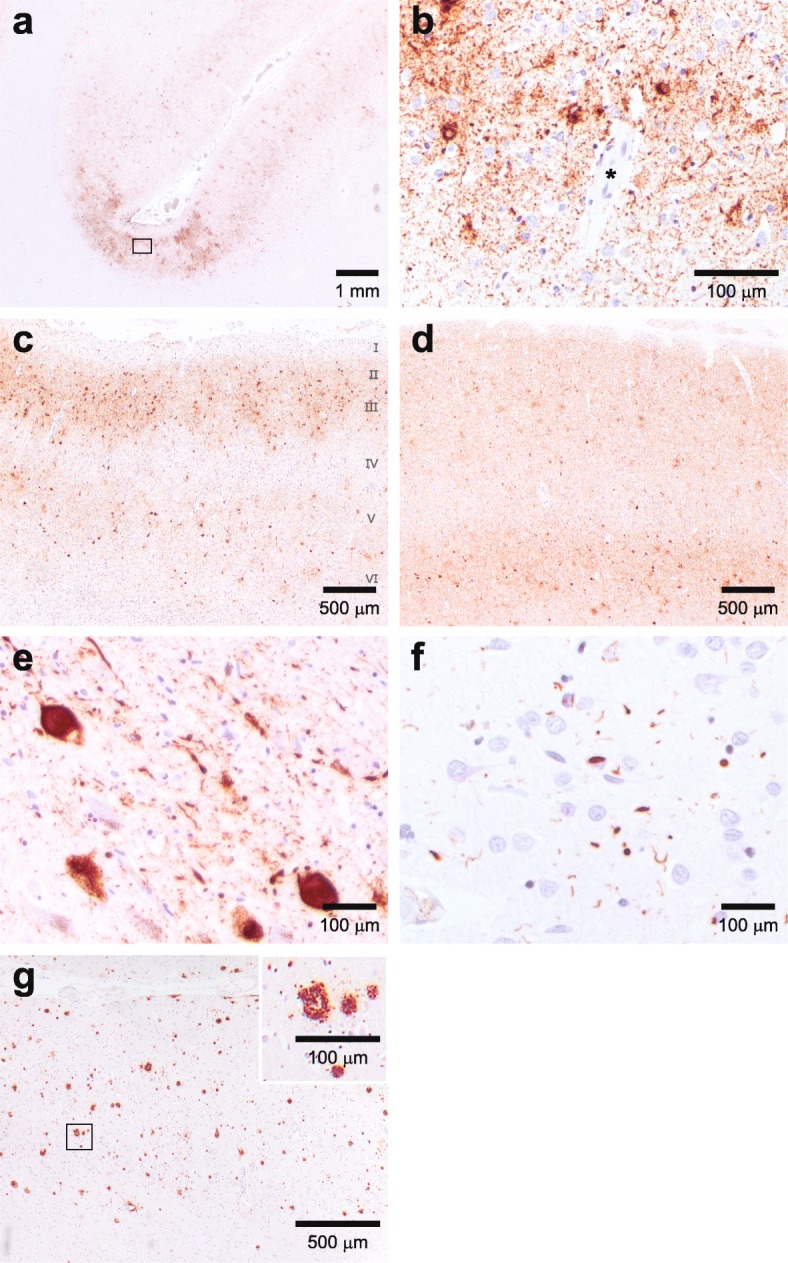
Immunohistochemical findings. **a**, **b** pTau (clone AT8, 1:800 dilution) immunoreactivity concentrated at the depths of a cortical sulcus in the superior frontal cortex (Brodmann area 8). pTau is found in the soma and processes of both neurons and astrocytes in an irregular distribution concentrated around blood vessels: the defining lesion of CTE. The boxed area in (**a**) is represented at high power in (**b**). **c** pTau staining of anterior superior temporal lobe (Brodmann area 38), showing dense immunoreactivity of both neurons and astrocytes concentrated in superficial cortical layers (layers II-III). This superficial pTau is more evenly distributed throughout temporal cortex, with only occasional denser foci at sulcal depths (four foci across four blocks of anterior temporal lobe). pTau is also present in deeper cortical layers as irregular/patchy clumps of mixed neuronal and astrocytic staining. **d** Inferior temporal gyrus from another individual (77yo ex-ARF player with AD but no CTE), showing a pattern of pTau pathology distinct to that of CTE, with neuronal pTau staining concentrated in deeper cortical layers and dense neuritic staining*.***e** Widespread pTau staining (as both globose tangles and pretangle pathology) in neurons of the substantia nigra, with accompanying neuritic pathology. There was accompanying moderate neuronal loss, pigment incontinence and gliosis. **f** pTDP-43 (clone 1D3, 1:500 dilution) staining of temporal lobe in the same superficial cortical layers depicted in (**c**), showing positive neuronal cytoplasmic inclusions and short neurites. **g** Beta-amyloid (betaA4 clone 6F/3D, 1:50 dilution) immunoreactivity in superior frontal cortex (Brodmann area 8)*.* The boxed area is represented at high power in the inset. All immunohistochemistry performed on 4 μm sections from standard-sized blocks of formalin-fixed (10% neutral buffered formalin), paraffin-embedded tissue on a Leica BOND-MAX™ autostainer using the Leica BOND Polymer Refine detection system as per the manufacturer’s recommendations

Hippocampal sclerosis was present, with some ghost tangles, gliosis, and heavy pTau involvement. Widespread neuronal and neuritic pTau was also present in amygdala, medial hypothalamic nuclei, mammillary body, nucleus basalis, substantia nigra (Fig. 1e), raphe nuclei and colliculi. Subpial and subependymal pTau in thorn-shaped astrocytes was present, consistent with aging-related tau astrogliopathy (ARTAG), most prominent in the temporal lobe.

Phosphorylated TDP-43 was present as neuronal cytoplasmic inclusions and short neurites, and was colocalised with regions of severe CTE pathology (Fig. 1f), a common finding in CTE [8]. Beta-amyloid and neuritic plaques were seen, corresponding to Thaal phase 4 (A3; Fig. 1g), and CERAD score of C2. While pTau pathology was in the typical distribution of CTE rather than AD, assessing all neurofibrillary tangle pathology gave a Braak stage of IV (B2). Together this equated to intermediate AD-neuropathologic change (A3,B2,C2) [6]. Immunohistochemistry for alpha-synuclein was negative.

Severe arteriolosclerosis was present in basal ganglia and white matter. Rarefaction and gliosis in subcortical white matter was generally mild-moderate, while in the anterior commissure and external capsule it was severe. Axonal pTau was moderate in these above two tracts, and mild elsewhere, and was seen as immunoreactive neurites and axonal varicosities. Beta-amyloid precursor protein was absent from anterior commissure and external capsule, and present in internal capsule in a pattern consistent with agonal changes only.

Taken together, these findings demonstrate severe (Stage III) CTE. This is the first confirmed case in ARF. CTE was associated with early-onset dementia, with neuropsychological features commonly described in pathologically confirmed CTE cases from other sports. Typical CTE pathology in this case was accompanied by intermediate AD-neuropathologic change, and severe small vessel disease.

ARF is the most popular contact sport in Australia, with a player base of more than 1.5 million, and a significant (30%) female representation. ARF is characterized by its fast-paced physicality: it involves running at speed, frequent jumping, and high-impact landing. With 18 players per side high-force collisions are commonplace, and can occur in any direction, on the ground or in the air. Thus unsurprisingly, ARF has a high injury and concussion rate [7], and the unique nature of the game places players at risk of head injury from multiple and complex mechanisms, distinct from those of the rugby codes. The limited available evidence on long-term neurological outcomes of ARF players suggests that, like ex-athletes of other contact sports, they too are predisposed to develop persisting deficits in motor control and cognition [4, 10].

There are no criteria for distinguishing AD-associated from CTE-associated pTau pathology when there is intercurrent disease. The identification of conformational differences in the β-helix region of pTau in CTE versus AD [5] suggests that these are two distinct pathologies, but currently *all* neurofibrillary tangle pathology in a CTE case is assessed to derive a Braak stage for AD. This ‘double-counting’ of pTau is likely to overestimate the severity of co-occurring AD in CTE, particularly in older individuals such as described here. Development of conformation-specific antibodies specific for CTE-pTau would greatly assist in distinguishing these two diseases.

This case represents only the second ARF player brain donated to the recently established Australian Sports Brain Bank [1], and the first to be diagnosed with CTE. While we can make no claims of CTE incidence in ARF based on this index case, the distinctive and severe pTau pathology is something we have not encountered in our busy clinical practice outside of ex-contact sports players [2]. That it exists at all should serve as a call to action to recognise and research CTE, and the very clear association with repetitive head injury. Claims of a lack of demonstrated ‘causality’ are unhelpful, and arguably irrelevant when assessing a public and occupational health issue such as CTE.

## Data Availability

N/A

## Abbreviations

AD: Alzheimer’s disease
ARF: Australian Rules football
ARTAG: aging-related tau astrogliopathy
CTE: chronic traumatic encephalopathy
